# Systemic TLR2 tolerance enhances central nervous system remyelination

**DOI:** 10.1186/s12974-019-1540-2

**Published:** 2019-07-27

**Authors:** Nicholas J. Wasko, Meghan Horne Kulak, Debayon Paul, Alexandra M. Nicaise, Stephen T. Yeung, Frank C. Nichols, Kamal M. Khanna, Stephen Crocker, Joel S. Pachter, Robert B. Clark

**Affiliations:** 1 Department of Immunology, UConn Health, Farmington, CT 06030 USA; 2 Department of Medicine, UConn Health, Farmington, CT 06030 USA; 3 Department of Neuroscience, UConn Health, Farmington, CT 06030 USA; 4 Department of Periodontology, UConn Health, Farmington, CT 06030 USA; 5 Present Address: Department of Microbiology, Perlmutter Cancer Center at NYU Langone School of Medicine, New York, NY 10016 USA

**Keywords:** TLR, TLR2, Tolerance, Remyelination, Multiple sclerosis, Microglia

## Abstract

**Background:**

Multiple sclerosis (MS) is a central nervous system (CNS) autoimmune disease characterized by both inflammatory demyelination and impaired remyelination. Studies indicate that Toll-like receptor 2 (TLR2) signaling contributes to both the inflammatory component and the defective remyelination in MS. While most MS therapeutics target adaptive immunity, we recently reported that reducing TLR2 signaling in innate immune cells by inducing TLR2 tolerance attenuates adoptively transferred experimental autoimmune encephalomyelitis. Given that previous reports suggest TLR2 signaling also inhibits myelin repair, the objective of this study was to assess how reducing TLR2 signaling through TLR2 tolerance induction affects CNS myelin repair*.*

**Methods:**

Chow containing 0.2% cuprizone was fed to male and female wild-type (WT) C57BL/6 mice or TLR2-deficient (TLR2^−/−^) mice for 5 weeks to induce demyelination. During a 2-week remyelination period following discontinuation of cuprizone, WT mice received either low dose TLR2 ligands to induce systemic TLR2 tolerance or vehicle control (VC). Remyelination was evaluated via electron microscopy and immunohistochemical analysis of microglia and oligodendrocytes in the corpus callosum. Statistical tests included 2-way ANOVA and Mann-Whitney *U* analyses.

**Results:**

Inducing TLR2 tolerance in WT mice during remyelination significantly enhanced myelin recovery, restoring unmyelinated axon frequency and myelin thickness to baseline levels compared to VC-treated mice. Mechanistically, enhanced remyelination in TLR2 tolerized mice was associated with a shift in corpus callosum microglia from a pro-inflammatory iNOS^+^ phenotype to a non-inflammatory/pro-repair Arg1^+^ phenotype. This result was confirmed in vitro by inducing TLR2 tolerance in WT microglia cultures. TLR2^−/−^ mice, without TLR2 tolerance induction, also significantly enhanced myelin recovery compared to WT mice, adding confirmation that reduced TLR2 signaling is associated with enhanced remyelination.

**Discussion:**

Our results suggest that reducing TLR2 signaling in vivo by inducing TLR2 tolerance significantly enhances myelin repair. Furthermore, the enhanced remyelination resulting from TLR2 tolerance induction is associated with a shift in corpus callosum microglia from a pro-inflammatory iNOS^+^ phenotype to a non-inflammatory/pro-repair Arg1^+^ phenotype. While deletion of TLR2 would be an impractical approach in vivo, reducing innate immune signaling through TLR2 tolerance induction may represent a novel, two-pronged approach for treating both inflammatory and myelin repair components of MS.

**Electronic supplementary material:**

The online version of this article (10.1186/s12974-019-1540-2) contains supplementary material, which is available to authorized users.

## Introduction

Multiple sclerosis (MS) is a progressive demyelinating disease of the central nervous system (CNS) characterized by immune-mediated damage to the myelin sheath surrounding neuronal axons [1]. Approximately 80% of cases present as relapsing-remitting MS (RRMS), with periodic clinical attacks punctuating periods of stability. Many such cases transition into secondary progressive MS (SPMS), in which neurological disability accumulates more consistently [1]. Alternatively, primary progressive MS (PPMS) occurs in 15% of cases, manifesting as continuous deterioration of neural function [2]. Immune-targeting treatments can reduce symptomatic flares in RRMS, but no existing therapies demonstrate consistent efficacy in treating SPMS or PPMS [3, 4]. The pathophysiology of MS includes loss of myelin-producing oligodendrocytes (OLs) [5], and while demyelination can theoretically be repaired by differentiation of oligodendrocyte precursor cells (OPCs) into mature OLs, for reasons as yet unknown, remyelination is defective in MS [5–8]. Diminished differentiation of OPCs into mature OLs may contribute to this defect in remyelination [3, 8, 9]. Enhancing remyelination has become an increasingly important goal of MS research.

Although the role of the adaptive immune system is well established in both MS and its murine model, experimental autoimmune encephalomyelitis (EAE), the role of the innate immune system has been less well explored. Studies have suggested that signaling through Toll-like receptor 2 (TLR2) plays a critical role in the inflammatory pathogenesis of MS and EAE [10–12], but only few studies have investigated a role for TLR2 signaling in remyelination. Of these, most have demonstrated an inhibitory effect of TLR2 signaling on remyelination [13–15].

Identifying the role of TLR2 signaling in the pathogenesis of MS is complicated by a potential role for the microbiome. One postulated function of the microbiome is to set the homeostatic threshold for TLR2 responsiveness through a relative “tolerance induction,” or regulation mediated by seeding the systemic circulation with low levels of TLR2 signaling microbial products [10, 16]. In proof-of-concept studies, we demonstrated that: a microbiome-derived bacterial lipopeptide, Lipid 654, can be detected in the serum of all healthy individuals but is found at significantly lower levels in the serum of MS patients [16]; induction of TLR2 tolerance via administration of low-dose TLR2 ligands results in the attenuation of adoptive transfer murine EAE [10]; and a significant proportion of MS patients demonstrate enhanced responsiveness to TLR2 stimulation [17].

Based on studies relating TLR2 to the inhibition of remyelination [13–15] and the potential for therapeutic intervention in EAE and MS via TLR2 tolerance induction [10, 17], the goal of the present study was to test the role of TLR2 and TLR2 tolerance induction in the process of CNS remyelination. Using the cuprizone model of demyelination, we now report that remyelination is enhanced when TLR2 signaling is diminished by the induction of TLR2 tolerance. These findings thus raise the possibility that this approach might ameliorate both the inflammatory and remyelinating aspects of MS pathogenesis.

## Methods

### Mice

Male and female C57BL/6 wild-type (WT) mice were purchased from Jackson Laboratory (Bar Harbor, ME). TLR2^−/−^ mice, bred onto a C57BL/6 background, were a generous gift of S. Akira (Osaka University, Japan). All mice were maintained under specific pathogen-free conditions in accordance with the guidelines for the Center for Comparative Medicine at UConn Health. All procedures were performed in compliance with Institutional Animal Care and Use Committee-approved protocols.

### Cuprizone demyelination model

Male or female wild-type (WT) C57BL/6 mice or TLR2^−/−^ mice (6–10 weeks old) were fed milled chow containing 0.2% bis (cyclohexanone) oxaldihydrazone (cuprizone; Sigma-Aldrich, St. Louis, MO), with food and water available ad libitum. Cuprizone feeding was maintained for 35 days to induce demyelination, then changed to normal rodent chow for the following 14 days to allow remyelination to occur.

### In vivo TLR2 tolerance induction

Starting on day 33, mice received intravenous (i.v.) injections of PBS vehicle control (VC) or Pam2CSK_4_ (P2C) (2.5 μg) (InvivoGen; San Diego, CA). Injections were given once per day for 5 days, then every other day through the remainder of the 14-day post-cuprizone recovery period.

### Assessment of in vivo TLR2 tolerance

To test for systemic TLR2 tolerance, on day 14 of the post-cuprizone recovery period, mice received an i.v. injection of 100 μg Pam3CSK_4_ (P3C; InvivoGen). Mice were bled 2 h later, and serum TNFα was assayed using Ready-SET-Go! ELISA kits (Affymetrix; Santa Clara, CA).

### Electron microscopy

On day 15 of the post-cuprizone recovery period, mice were anesthetized with ketamine and perfused transcardially with 5 mL of PBS followed by 10 mL of cold 2% paraformaldehyde (PFA)/2.5% glutaraldehyde. Brains were removed, stored overnight at 4 °C in 2% PFA/2.5% glutaraldehyde, then the corpus callosum (CC) was dissected out and transferred to 0.1 M cacodylate buffer. This tissue was then post-fixed in OsO_4_, dehydrated in ethanol, and embedded in epoxy resin. Ultrathin 70-nm sections were stained with uranyl acetate and Sato’s Lead citrate and examined with a Hitachi H-7650 TEM.

### Assessment of myelination

Digitized, non-overlapping electron micrographs of the CC were analyzed for unmyelinated axon frequency and g-ratios. To calculate the unmyelinated axon frequency, 300+ axons from representative images (500 μm^2^ per animal) were analyzed. Unmyelinated axons were counted as those with a total absence of surrounding myelin. For g-ratio analysis, a minimum of 100 randomly selected axons were measured using a plug-in for the ImageJ software which allowed for semi-automated analysis of randomly selected sets of fibers [18]. Fibers with prominent outfoldings in the plane of section were excluded.

### Tissue section preparation

On day 15 of the post-cuprizone recovery period, mice were anesthetized with ketamine and perfused transcardially with 5 mL of PBS followed by 10 mL of cold 4% PFA. Brains were removed, post-fixed overnight in 4% PFA, then cryoprotected in 30% sucrose. Coronal sections of 40 μm thickness (Bregma + 1.10 mm to − 2.30 mm) were cut using a Microm HM440E microtome. Sections were stored at 4 °C in PBS containing 0.05% sodium azide until stained.

### Immunohistofluorescence

Tissue sections were blocked and permeabilized at room temperature for 1 h in 5% bovine serum albumin (Sigma), 0.05% Triton X-100 (Sigma), and 5% heat-inactivated donkey serum (Millipore) or goat serum (Millipore) for microglia or OPC/OL staining, respectively. Antibodies for microglia stains were 1:1,000 rabbit anti-IBA1 (Wako; Richmond, VA), 1:200 mouse anti-iNOS (Invitrogen; San Diego, CA), 1:50 goat anti-Arg1 (NovusBio; Littleton, CO), 1:500 donkey anti-rabbit IgG Alexafluor 546 (Invitrogen), 1:500 donkey anti-mouse IgG Alexa-Fluor 647 (Invitrogen), and 1:250 donkey anti-goat IgG Alexa-Fluor 488 (Jackson Labs). Antibodies for OPC/OL stains were 1:250 rabbit anti-Olig2 (NovusBio), 1:500 mouse anti-APC, clone CC1 (Millipore), 1:500 rat anti-PDGFRα (Invitrogen), 1:500 goat anti-rabbit IgG Alexa-Fluor 647 (Invitrogen), 1:1,000 goat anti-mouse IgG Alexa-Fluor 546 (Invitrogen), and 1:2,000 goat anti-rat IgG Alexa-Fluor 488 (Abcam; Cambridge, MA). Primary antibodies were incubated overnight at 4 °C and secondary antibodies were incubated for 2 h at room temperature. Nuclei were stained with 1:1,000 DAPI (Thermo Fisher Scientific; Waltham, MA) for 10 min at room temperature. Slides were mounted with Mowiol (Sigma), imaged with a Zeiss LSM 880 confocal microscope, acquired using Zen Black software, and analyzed on Bitplane IMARIS software (Concord, MA).

### In vitro microglia TLR2 tolerance

Whole brain cultures were established from P1-P3 WT mouse pups and plated in T75 flasks as described previously [19]. The culture media was removed and replaced twice per week for approximately 10–15 days. When adherent astrocytes were confluent for at least 5 days, non-adherent cells were harvested and assessed for microglial purity by flow cytometry after labeling with antibodies to CD45.2 and CD11b. In 4 of 6 studies, non-adherent cells were further purified by FACS-sorting for CD45.2^+^ CD11b^+^ microglia (Additional file 1: Fig. S1). Non-adherent cells (65–75% CD45.2^+^ CD11b^+^), or FACS-sorted microglia (> 99% CD45.2^+^ CD11b^+^), were plated at 2–7 × 10^4^ cells/well in 96-well plates pre-coated with poly-L Lysine (0.1 mg/mL). Ninety-six-well microglial cultures were designated as either “non-stimulated,” “stimulated,” or “tolerized.” “Non-stimulated” wells received no stimulation over the ensuing entire 2 days of culture. “Stimulated” wells received 1 μg/mL P2C at the 24 hour time point. “Tolerized” wells received 1 μg/mL P2C both at the initiation of culture and at the 24 hour time point. After 48 hours, supernatants were harvested and assayed for cytokines via multiplex enzyme-linked immunosorbent assay (ELISA).

### Statistical analysis

**Fig. 1:**
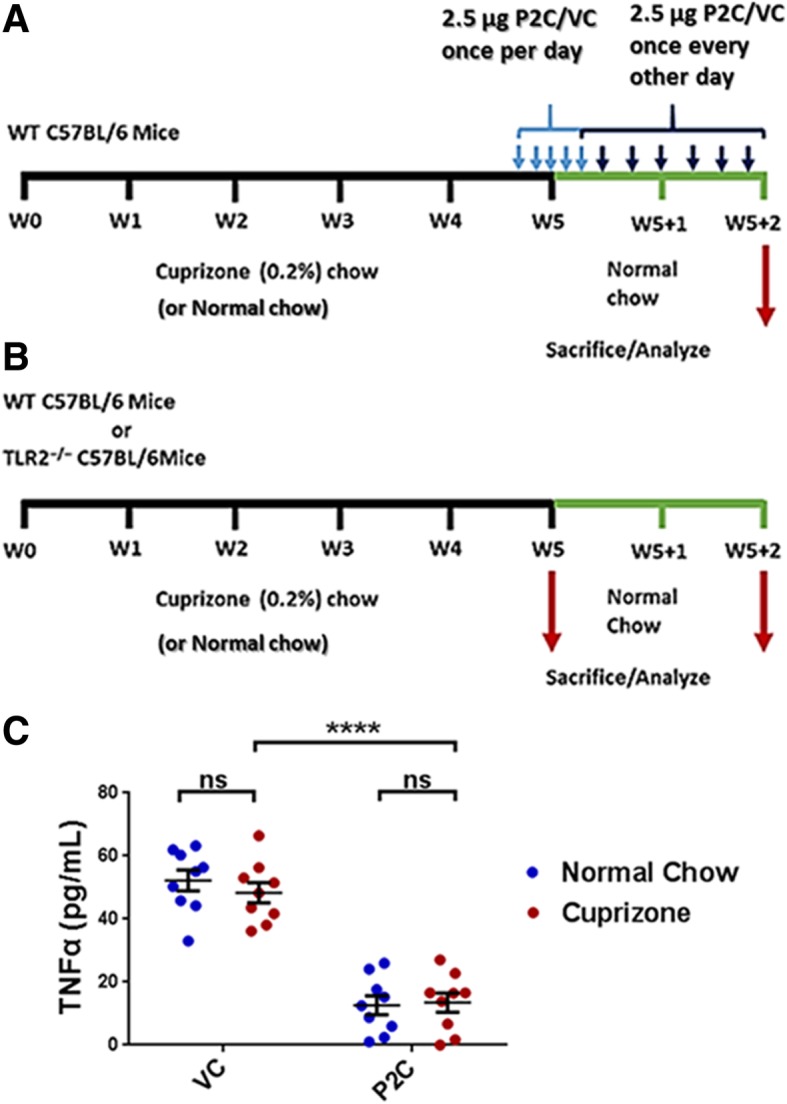
**a** Timeline of TLR2 tolerance induction. Timeline for the 5-week induction of demyelination with 0.2% cuprizone (or normal chow), followed by the 2-week remyelination period on normal chow. WT mice were treated with either low dose P2C or VC during the remyelination period. Mice were sacrificed and analyzed after the 2-week remyelination period. **b** Timeline of TLR2^−/−^. Timeline for the 5-week induction of demyelination with 0.2% cuprizone (or normal chow) followed by the 2-week remyelination period. WT and TLR2^−/−^ mice were not treated during the remyelination period. Mice were sacrificed and analyzed immediately following the 5-week cuprizone (or normal chow) feeding to evaluate baseline demyelination levels, or after the 2-week remyelination period on normal chow to evaluate myelin recovery. **c** Systemic TLR2 tolerance induction. At the end of the 2-week remyelination period, WT mice that had been fed for the first 5 weeks with either cuprizone or normal chow and treated with P2C or VC as in **a** received a single i.v. injection of Pam3CSK_4_ (P3C, 100 μg) and 2 h later serum was obtained and analyzed for TNFα by ELISA. Results are expressed as mean values per mouse. *N* = 9 mice/cohort. Error bars represent the mean ± SEM. Statistical differences were assessed by 2-way ANOVA. ****p < 0.0001. For NS comparisons, VC treated: normal vs cuprizone-fed, *p* = 0.8118. P2C treated: normal vs cuprizone-fed, *p* = 0.9972

**Fig. 2:**
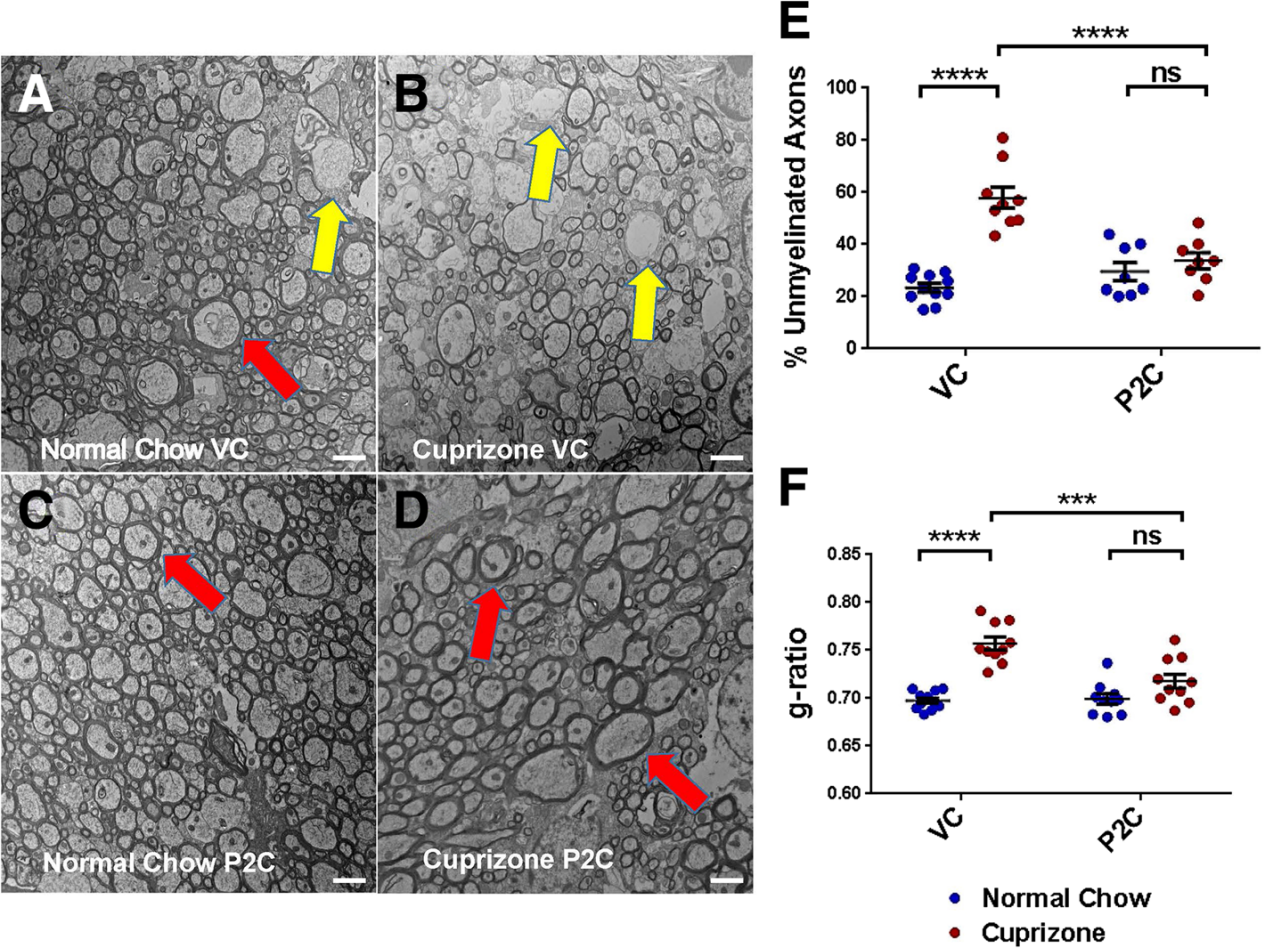
Effect of TLR2 tolerance induction on remyelination. **a**–**d** Representative EM images from corpus callosum: **a** “Normal Chow VC”—mice fed for 7 weeks with normal chow and treated for the last 2 weeks with VC. **b** “Cuprizone VC”—mice fed for 5 weeks with cuprizone, then fed for 2 weeks with normal chow and treated for these last 2 weeks with VC. **c** “Normal Chow P2C”—mice fed for 7 weeks with normal chow and treated for the last 2 weeks with Pam2CSK_4_ (P2C). **d** “Cuprizone P2C”—mice fed for 5 weeks with cuprizone, then fed for 2 weeks with normal chow and treated for these last 2 weeks with P2C. Scale bar = 2 μm. Red arrows depict typical axons with normal myelin thickness. Yellow arrows depict typical axons with decreased myelin thickness. **e** Percentage of unmyelinated axons as calculated from EM images. **f** Myelin thickness (g-ratios) as calculated from EM images. For **e** and **f**, data points represent individual mice analyzed as described in the “Methods” section. *N* = 8–10 mice per experimental condition. Error bars represent the mean ± SEM. Statistical differences were assessed by 2-way ANOVA. ***p < 0.001; ****p < 0.0001. For NS comparisons: P2C treated: normal vs cuprizone-fed, *p* = 0.7912 (**e**). P2C treated: normal vs cuprizone-fed, *p* = 0.1486 (**f**)

**Fig. 3:**
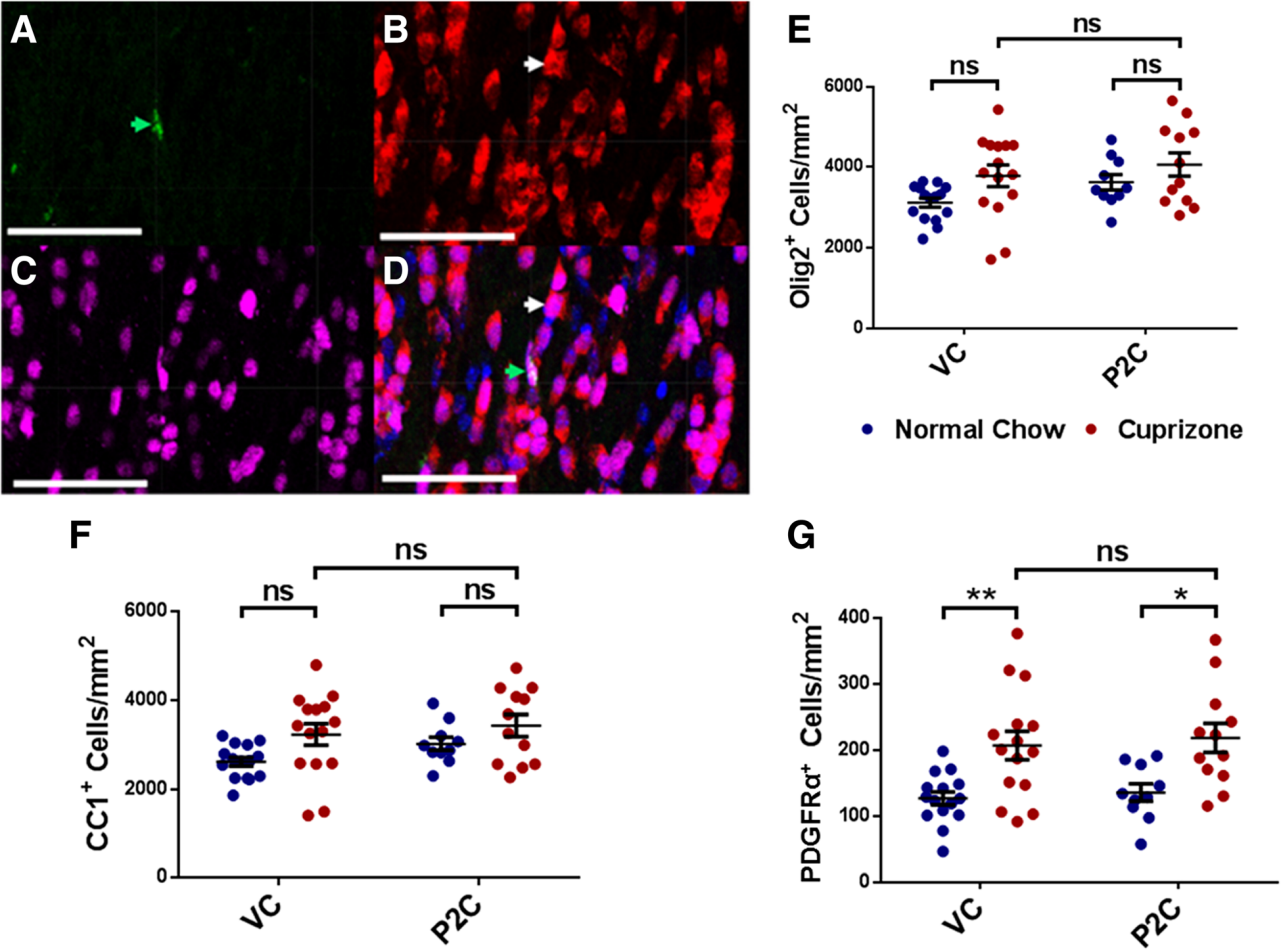
Effect of TLR2 tolerance induction on OPC and OL frequency. **a**–**d** Overlapping immunohistofluorescent stains of one representative corpus callosum section. **a** Green = PDGFRα^+^ cells, **b** red = CC1^+^ cells, **c** magenta = Olig2^+^ cells, **d** all images merged; blue = DAPI. In **d**, the white arrow depicts the example CC1^+^ cell also seen in **b**, and the green arrow depicts the example PDGFRα^+^ cell also seen in **a**. Scale bars represent 50 μm. **e–g** Analysis of cell frequency in corpus callosum sections based on co-localization of DAPI^+^ Olig2^+^ cells/mm^2^. **e** Olig2^+^ cells/mm^2^, **f** Olig2^+^ CC1^+^ cells/mm^2^, **g** Olig2^+^ PDGFRα^+^ cells/mm^2^. Data points represent individually analyzed sections derived from 4 mice per cohort; 3–4 sections were analyzed from each mouse. *N* = 12–15 total sections per experimental cohort. Error bars represent the mean ± SEM. Statistical differences were assessed by 2-way ANOVA. *p < 0.05; **p < 0.01. For NS comparisons: **e** VC treated: normal vs cuprizone-fed, *p* = 0.1660. P2C treated: normal vs cuprizone-fed, *p* = 0.7672. Normal chow: VC vs P2C, *p* = 0.5803. Cuprizone-fed: VC vs P2C, *p* = 0.9451. **f** VC treated: normal vs cuprizone-fed, *p* = 0.1295. P2C treated: normal vs cuprizone-fed, *p* = 0.7142. Normal chow: VC vs P2C, *p* = 0.6692. Cuprizone-fed: VC vs P2C, *p* = 0.9772. **g** Cuprizone-fed: VC vs P2C, *p* = 0.9983

**Fig. 4:**
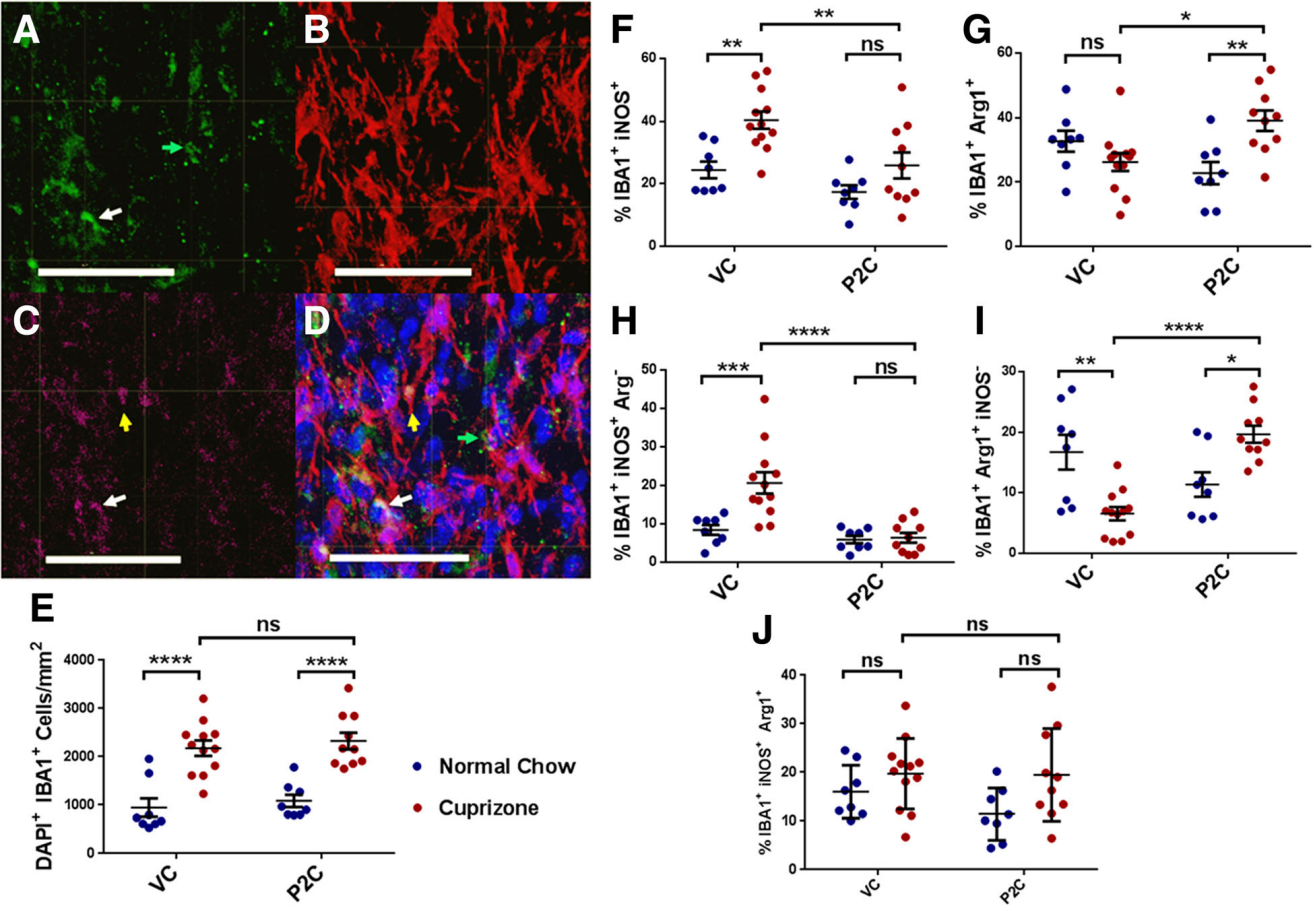
Effect of TLR2 tolerance induction on microglia frequency and phenotype. **a**–**d** overlapping immunohistofluorescent stains of one representative corpus callosum section. **a** green = Arg1^+^ cells, **b** red = IBA1^+^ cells; **c** magenta = iNOS^+^ cells; **d** all images merged; blue = DAPI. In **d**, the green arrow depicts the example Arg1^+^ cell also shown in **a**, the yellow arrow depicts the example iNOS^+^ cell also seen in **c**, and the white arrow depicts the example double-positive, iNOS^+^Arg1^+^ cell, also seen in **a** and **c**. Scale bars represent 50 μm. **e** Analysis of IBA1^+^ cell frequency in corpus callosum sections, based on co-localization of DAPI^+^ IBA1^+^ cells. **f** Percentage of IBA1^+^ cells that are iNOS^+^ (total = single and double positives). **g** Percentage of IBA1^+^ cells that are Arg1^+^ (total = single and double positives). **h** Percentage of IBA1^+^ cells that are iNOS^+^ single positive (Arg1^−^). **i** Percentage of IBA1^+^ cells that are Arg1^+^ single positive (iNOS^−^). **j** Percentage of IBA1^+^ cells that are iNOS^+^Arg1^+^ double-positive. Data points represent individually analyzed sections derived from 4 mice per cohort; 2–3 sections were analyzed from each mouse. *N* = 8–12 total sections per experimental cohort. Error bars represent the mean ± SEM. Statistical differences were assessed by 2-way ANOVA, *p < 0.05; **p < 0.01; ***p < 0.001; ****p < 0.0001. For NS comparisons: **e** Cuprizone-fed: VC vs P2C, *p* = 0.9042. **f** P2C treated: normal vs cuprizone-fed, *p* = 0.3729. **g** VC treated: normal vs cuprizone-fed, *p* = 0.6316. **h** P2C treated: normal vs cuprizone-fed, *p* > 0.9999. **j** VC treated: normal vs cuprizone-fed, *p* = 0.6881. P2C treated: normal vs cuprizone-fed, *p* = 0.1123. Cuprizone-fed: VC vs P2C, *p* = 0.9999

**Fig. 5:**
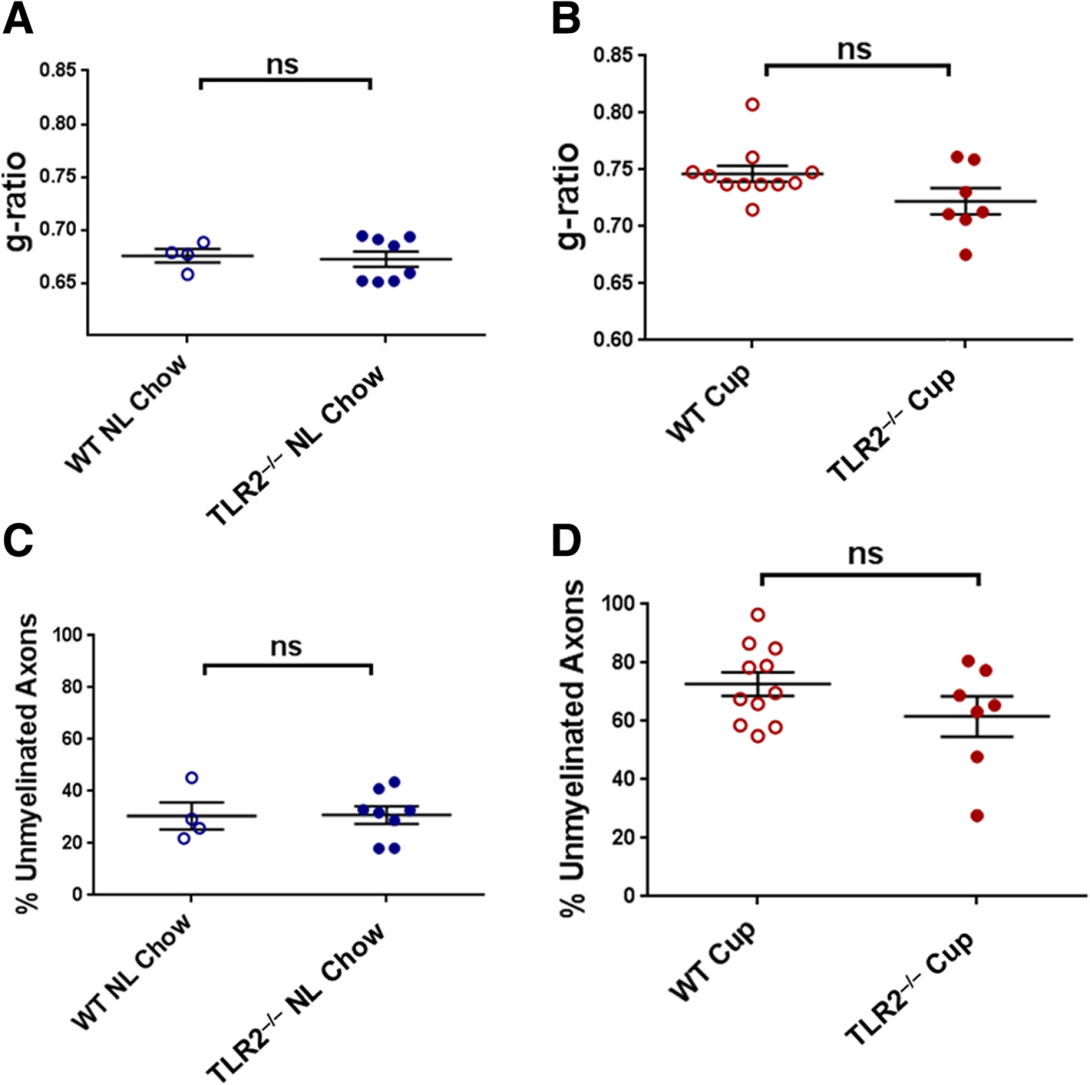
Demyelination in WT versus TLR2^−/−^ mice after 5 weeks on cuprizone. WT and TLR2^−/−^ mice were fed either cuprizone or normal chow for 5 weeks, sacrificed, and myelin status in the corpus callosum was analyzed by EM. **a** Myelin thickness (g-ratios) of normal (“NL”) chow-fed WT versus TLR2^−/−^ mice. **b** Myelin thickness of cuprizone (“Cup”)-fed WT versus TLR2^−/−^ mice. **c** Percentage of unmyelinated axons in normal chow-fed WT versus TLR2^−/−^ mice**. d** Percentage of unmyelinated axons in cuprizone-fed WT versus TLR2^−/−^ mice. Data points represent individual mice analyzed as described in Methods. *N* = 4–11 mice per experimental condition. Error bars represent the mean ± SEM. Statistical differences were assessed by unpaired *t* test with Welch’s correction or Mann-Whitney analysis. For NS comparisons: **a** NL chow: WT vs TLR2^−/−^, *p* > 0.9999. **b** Cuprizone-fed: WT vs TLR2^−/−^, *p* = 0.1020. **c** NL chow: WT vs TLR2^−/−^, *p* = 0.9660. **d** Cuprizone-fed: WT vs TLR2^−/−^, *p* = 0.1941

**Fig. 6:**
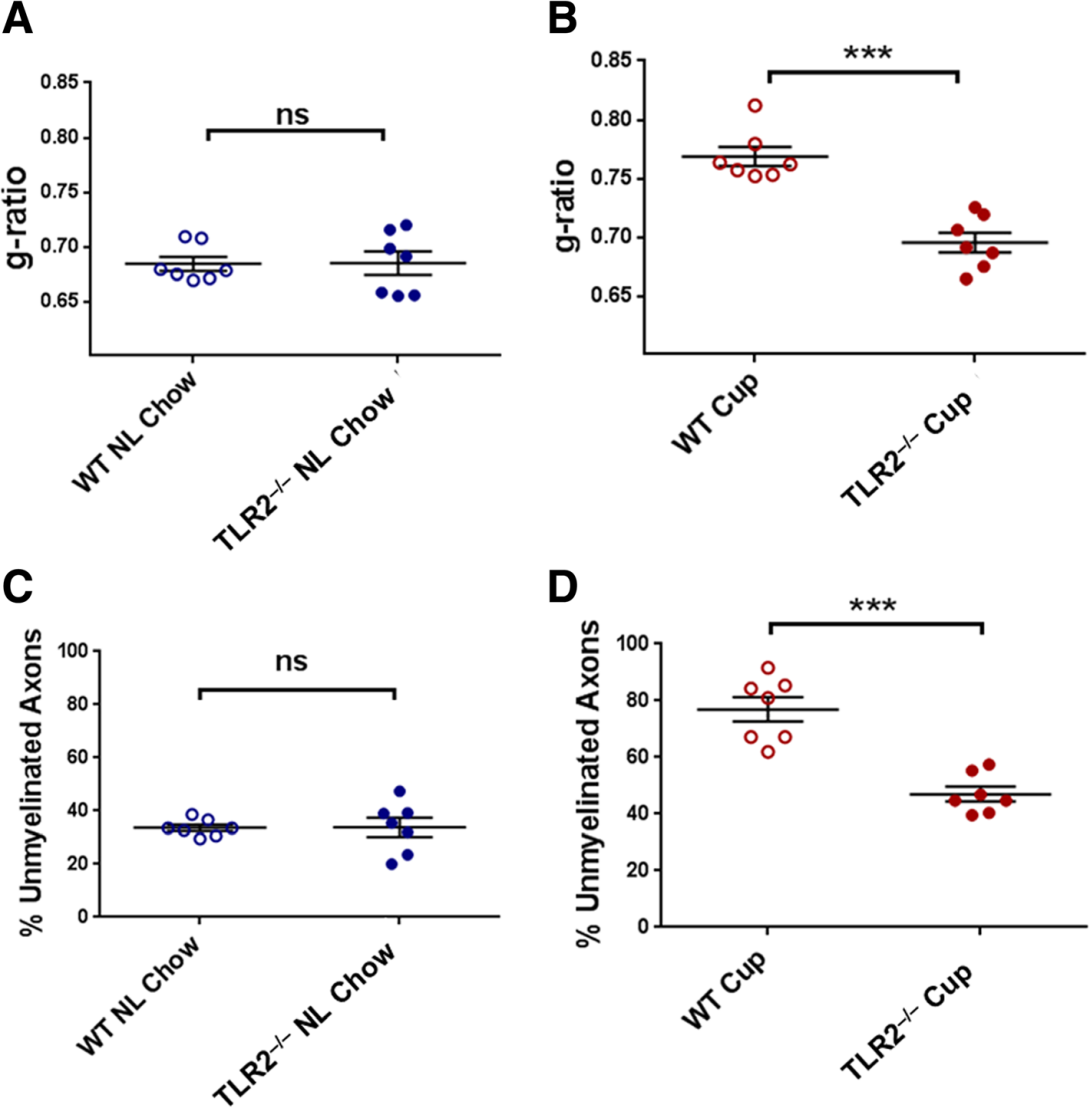
Demyelination in WT versus TLR2^−/−^ mice after 5 weeks on cuprizone and 2 weeks on normal chow. WT and TLR2^−/−^ mice were fed either cuprizone or normal chow for 5 weeks, then all mice were fed normal chow for 2 weeks (“recovery period”). At the end of the 2-week recovery period, myelin status in the corpus callosum was analyzed by EM. **a** Myelin thickness (g-ratios) of normal (“NL”) chow-fed WT versus TLR2^−/−^ mice. **b** Myelin thickness of cuprizone (“Cup”)-fed WT versus TLR2^−/−^ mice. **c** Percentage of unmyelinated axons in normal chow-fed WT versus TLR2^−/−^ mice. **d** Percentage of unmyelinated axons in cuprizone-fed WT versus TLR2^−/−^ mice. Data points represent individual mice analyzed as described in the “Methods” section. *N* = 7 mice per experimental condition. Error bars represent the mean ± SEM. Statistical differences were assessed by Mann-Whitney analysis. ***p < 0.001. For NS comparisons: **a** NL chow: WT vs TLR2^−/−^, *p* = 0.9656. **c** NL chow: WT vs TLR2^−/−^, *p* = 0.9636

**Fig. 7:**
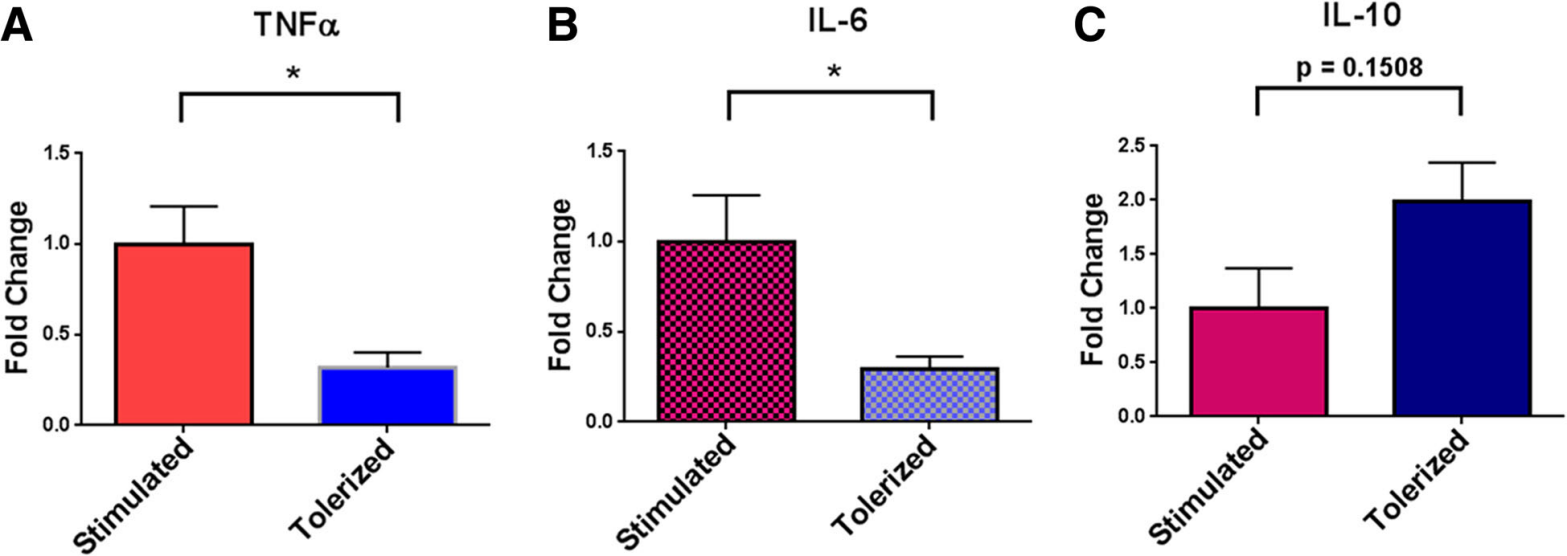
In vitro microglia TLR2 tolerance. Mixed glial cultures were established from P1–P3 WT mouse pups. After 12–18 days in culture, non-adherent cells (microglia) were harvested and either directly plated for testing in 96-well plates or plated after further purification by FACS-sorting for CD45.2^+^ CD11b^+^ microglia. Microglia were plated at 2–7 × 10^4^ cells/well in 96-well plates pre-coated with poly-L lysine. Microglial cultures were designated as either “non-stimulated,” “stimulated,” or “tolerized”. “Non-stimulated” wells received no stimulation over the entire 48 h in culture. “Stimulated” wells received 1 μg/mL P2C only at the 24 h point of culture. “Tolerized” wells received 1 μg/mL P2C both at the initiation of culture (hour 0) and again at the 24 h point of culture. After 48 h, supernatants were harvested and assayed for cytokines via multiplex ELISA. Cytokine levels in “non-stimulated” culture supernatants were either very low or below the level of detection (see Additional file 2: Table S1). Results are expressed as fold change of cytokine levels in “stimulated” versus “tolerized” culture supernatants for **a** TNFα; **b** IL-6; **c** IL-10. *N* = 6 total experiments; 2 using non-FACS-sorted microglia (65–75% CD45^+^ CD11b^+^ cells) and 4 using FACS-sorted microglia. Error bars represent the mean ± SEM. Statistical differences were assessed by Mann-Whitney analysis. *p < 0.05

Figures 1c, 2, 3, and 4 utilized 2-way ANOVA, with values below *p* = 0.05 considered significant. Figures 5, 6, and 7 utilized Mann-Whitney *U* analyses, with values below *p* = 0.05 considered significant.

## Results

### In vivo induction of TLR2 tolerance

To assess the effect of TLR2 tolerance on myelin repair, we utilized the cuprizone model of demyelination. WT mice were fed chow containing cuprizone for 5 weeks to induce demyelination, then given a 2-week remyelination (“recovery”) period during which time they were fed normal chow (Fig. 1a). Alternatively, WT mice received normal chow for the entire 7 week period. Remyelination was then assessed in the CC of all cohorts at the end of the 2-week recovery period.

It is well documented both in vitro and in vivo that stimulation with a TLR ligand can induce significant downregulation of pro-inflammatory responses upon subsequent TLR ligation (“tolerance induction”) [20]. In this case, TLR2 tolerance was induced in the 5-week cuprizone/2-week normal chow cohort as well as in the 7-week normal chow cohort, starting on the last 2 days of week 5 and continuing through the 2-week “recovery” period. TLR2 tolerance was induced by repeated administration of a low dose of the TLR2 ligand Pam2CSK_4_ (P2C); 2.5 μg of P2C was given i.v. once per day for 5 days, then every other day for the remaining 12 days of the 14-day recovery period. Control cohorts of mice on the 5-week cuprizone/2-week normal chow schedule, as well as mice on the 7-week normal chow schedule, received vehicle control (VC) i.v. rather than P2C (Fig. 1a).

At the end of the 2-week recovery period, systemic TLR2 tolerance was assessed by challenging mice with a single i.v. injection of a large dose (100 μg) of a different TLR2 ligand, Pam3CSK_4_ (P3C). The systemic response to the P3C challenge was analyzed by obtaining serum samples 2 h later and measuring serum TNFα levels by ELISA. Mice treated with VC, regardless of whether they had been on the 5-week cuprizone/2 week normal chow schedule or the 7-week normal chow schedule, showed a strong systemic response to the P3C challenge as indicated by high levels of serum TNFα (Fig. 1c). In contrast, mice treated with P2C throughout the recovery period, regardless of prior feeding schedule, demonstrated a significantly reduced systemic TNFα response to the P3C challenge (Fig. 1c). These results indicate that repeated low-dose P2C injections during the recovery period induced a state of systemic TLR2 tolerance, with a significant decrease in the ligand-activated systemic TLR2 pro-inflammatory response.

### TLR2 tolerance promotes remyelination following cuprizone-induced demyelination

To assess the efficiency of remyelination following 5 weeks of cuprizone and 2 weeks of recovery, mice were sacrificed and myelination in the CC analyzed by electron microscopy (EM). Electron micrographs of CC cross sections were evaluated for the percentage of myelinated fibers and thickness of myelin sheaths (i.e., g-ratio). Among the VC-treated mice, those fed with cuprizone demonstrated a significantly higher percentage of axons lacking myelin (“unmyelinated”) compared to those fed with normal chow for the first 5 weeks (Fig. 2a, b, e). In contrast, among the P2C treated/TLR2-tolerized mice, those fed with cuprizone showed no significant increase in the percentage of unmyelinated axons compared to those fed with normal chow (Fig. 2c–e). These results suggest that inducing TLR2 tolerance during the recovery period restored the frequency of unmyelinated axons to baseline levels (Fig. 2e).

In addition to measuring the percentage of myelinated fibers, myelin thickness was measured using EM analysis of g-ratios. G-ratios were calculated as the ratio of the inner axonal diameter to the total outer diameter. Among VC-treated mice, those fed with cuprizone demonstrated a significantly higher g-ratio (i.e., less myelin thickness) than those fed with normal chow (Fig. 2a, b, f). In contrast, among P2C/TLR2-tolerized mice, those fed with cuprizone showed no significant increase in g-ratio (i.e., no thinning of myelin) compared to those fed with normal chow (Fig. 2c, d, f). These data suggest that inducing TLR2 tolerance during the recovery period restored the myelin thickness to baseline levels (Fig. 2f).

### TLR2 tolerance does not increase OPC or OL frequency following cuprizone-induced demyelination

It has been reported that OPCs express TLR2 and that ligation of TLR2 can have an inhibitory effect on OPC maturation to mature OLs [15]. To determine whether the improved rate of myelin repair found in P2C-treated/TLR2-tolerized mice reflected increased numbers of OPCs or increased maturation of OPCs into myelin-producing OLs, mice treated either with VC or P2C during the 2-week recovery period were evaluated using an immunohistofluorescence approach. CC sections were stained with anti-Olig2 antibodies, which delineated all cells of OL lineage, anti-PDGFRα antibodies to label OPCs, or anti-APC (CC1 clone) antibodies to label mature OLs. Representative stains are shown in Fig. 3a–d. We then analyzed the absolute numbers of Olig2^+^ cells, the absolute numbers of Olig2^+^ CC1^+^ mature OL cells, and the absolute numbers of Olig2^+^ PDGFRα^+^ OPCs.

Although not statistically significant, cuprizone-fed mice exhibited slightly higher numbers of Olig2^+^ cells than mice that received only normal chow, which was seen in both VC- and P2C-treated cohorts (Fig. 3e). Absolute numbers of CC1^+^ mature OLs showed a similar trend, which also did not differ significantly between VC and P2C-treated cohorts (Fig. 3f). Cuprizone-fed mice did demonstrate a significant increase in PDGFRα^+^ OPCs compared to mice that received normal chow. However, this increase in PDGFRα^+^ OPCs was present and equal in both VC and P2C-treated cohorts (Fig. 3g). These results indicate that inducing TLR2 tolerance with P2C did not significantly alter the absolute numbers of OPCs or mature OLs compared to VC-treated mice (Fig. 3f, g). This suggests that the enhancement of remyelination seen in P2C-treated/TLR2-tolerized mice is not mediated through direct effects on OPC numbers or on their maturation into OLs.

### TLR2 tolerance decreases iNOS^+^ microglia and increases Arg1^+^ microglia following cuprizone-induced demyelination

Microglia represent an important regulator of CNS myelination and given their well-documented expression of TLR2 we next investigated their relevance in TLR2 tolerance-induced enhancement of remyelination [21–23]. We postulated that TLR2 tolerance induction would result in an enhancement of the microglial non-inflammatory/pro-repair repair Arg1^+^ phenotype, known to be associated with enhanced remyelination [24–26].

To investigate this possibility, after the 2-week recovery period, CC sections were stained with antibodies to IBA1 to label all microglia, antibodies to iNOS to label microglia with a pro-inflammatory phenotype, and antibodies to Arg1 to label microglia with a non-inflammatory/pro-repair phenotype, markers known to correlate with pro- and anti-inflammatory functions, respectively, in the CNS [26–28]. Representative stains are shown in Fig. 4a–d. Sections were evaluated for the percentage of IBA1^+^ microglia which stained positive for either iNOS or Arg1 alone (“single positives”), as well as the percentage of microglia staining with both markers (“double positives”). The percentages of single iNOS- or Arg1-positive microglia were each added to the percentages of double-positive microglia to derive “total” iNOS- or “total” Arg1-positive microglia.

Cuprizone-fed mice demonstrated a significant microgliosis when compared to mice fed only with normal chow, and the increased number of microglia per square millimeter was the same regardless of VC or P2C treatment (Fig. 4e). Analysis of phenotypic staining revealed mice that were cuprizone-fed and treated with VC during the recovery period exhibited a significantly higher percentage of both single-positive and total iNOS^+^ microglia than VC-treated mice fed only with normal chow (Fig. 4h, f). These results suggest that cuprizone feeding induces an inflammatory phenotype in CC microglia. In contrast, mice that were cuprizone-fed but treated with P2C did not demonstrate this enhancement in the percentage of either single-positive or total iNOS^+^ microglia (Fig. 4h, f). This suggests that P2C treatment/TLR2 tolerance induction mitigated this cuprizone-induced inflammatory microglial phenotype.

Next, the percentage of Arg1^+^ microglia was analyzed in each cohort. In contrast to the increased percentage of iNOS^+^ microglia in cuprizone-fed VC-treated mice, such mice exhibited a significant decrease in the percentage of single-positive Arg1^+^ microglia when compared to normal chow-fed VC-treated mice (Fig. 4i). A similar decrease in the percentage of total Arg1^+^ microglia was also noted among cuprizone-fed VC-treated mice, but did not reach statistical significance (Fig. 4g). These results suggest that cuprizone feeding not only enhances the percentages of pro-inflammatory iNOS^+^ microglia but also decreases the percentages of non-inflammatory/pro-repair repair Arg1^+^ microglia.

A strikingly opposite trend was found among cuprizone-fed, P2C-treated mice. Rather than a significant decrease in the percentage of Arg1^+^ microglia, the cuprizone-fed, P2C-treated/TLR2-tolerized mice demonstrated a significantly higher percentage of both single-positive and total Arg1^+^ microglia (Fig. 4i, g, j). The iNOS^+^Arg1^+^ double-positive microglia were not significantly different across groups (Fig. 4j), indicating that the switch from single positive iNOS^+^-polarized to single positive Arg1^+^-polarized microglia was the primary driver behind this phenotypic change. In sum, the microglial analyses indicate that TLR2 tolerance induction during the post-cuprizone recovery phase results in a major microglial phenotypic switch, with a significant decrease in the percentage of iNOS^+^ pro-inflammatory microglia and a simultaneous increase in the percentage of Arg1^+^ non-inflammatory microglia.

### TLR2^−/−^ mice exhibit enhanced remyelination after cuprizone feeding

To confirm that the enhanced remyelination in P2C-treated mice was a result of TLR2 tolerance induction and not related to repeated low dose TLR2 stimulation, remyelination studies were performed utilizing TLR2-deficient (TLR2^−/−^) mice. Wild-type (WT; C57Bl/6) and TLR2^−/−^ mice (C57Bl/6 background) were fed for 5 weeks with either normal chow or cuprizone and then given a 2-week period on normal chow to allow for remyelination. In these studies, mice were not treated with either VC or P2C during the 2-week recovery period (Fig. 1b).

To determine if WT and TLR2^−/−^ mice differ in the degree of baseline demyelination mediated by 5 weeks of cuprizone exposure, normal chow-fed and cuprizone-fed mice were analyzed immediately after the initial 5-week demyelination period (Fig. 1b). EM analysis confirmed that neither unmyelinated axon frequency nor myelin thickness differed between WT and TLR2^−/−^mice fed normal chow for 5 weeks (Fig. 5a, c). Importantly, these parameters also did not differ between WT and TLR2^−/−^ mice fed cuprizone for 5 weeks (Fig. 5b, d). Thus, TLR2^−/−^ mice were not more or less susceptible than WT mice to cuprizone-mediated demyelination, consistent with previous reports [13].

Next, WT and TLR2^−/−^ mice were analyzed after 5 weeks of cuprizone or normal chow followed by a 2-week recovery period on normal chow. Similarly, myelin parameters did not differ between WT and TLR2^−/−^ mice fed normal chow for the entire 5 and 2 week periods (Fig. 6a, c). In contrast, after cuprizone feeding and a 2-week recovery period on normal chow, TLR2^−/−^ mice exhibited significantly enhanced recovery of myelinated axon frequency and myelin thickness when compared to WT mice (Fig. 6b, d). These results are consistent with earlier findings [15], suggesting that endogenous TLR2 signaling inhibits myelin repair. Moreover, these results are consistent with our conclusion that enhanced myelin repair following repeated low-dose P2C treatment is mediated by TLR2 tolerance induction rather than repeated TLR2 stimulation.

### In vitro TLR2 tolerance induction in microglia

Previous studies reported in vitro induction of TLR2 tolerance in bone marrow-derived macrophages (BMDMs), showing that TLR tolerance can be observed in vitro even after a single round of TLR stimulation [10, 20]. To further examine the effect of TLR2 tolerance on microglia, cultures of primary microglia were derived and tolerized in vitro using P2C. Primary microglia were isolated from cultures of whole mouse pup brains and, in some experiments, were FACS-sorted for purity*.* The isolated microglia were re-cultured in 96-well plates and remained either non-stimulated for 48 h, stimulated by the addition of 1 μg/mL P2C at 24 h of culture (“stimulated protocol”), or stimulated by the addition of 1 μg/mL P2C at both the initiation of culture (hour 0) and 24 h of culture (“tolerance protocol”). After 48 h, culture supernatants were harvested and assayed for levels of tumor necrosis factor α (TNFα), interleukin 6 (IL-6), and interleukin 10 (IL-10) by ELISA.

Two studies were performed in which microglia were not purified by FACS-sorting. In these studies, the purity of the isolated microglia (CD45^+^ CD11b^+^) was 65–75%. Four studies were performed in which microglia were isolated from cultures and then FACs-sorted. In these, the microglia purity was > 99% (Additional file 1: Fig. S1). The results of the 48 h testing of microglial stimulation and tolerance did not differ significantly between studies that used non-sorted versus sorted microglia, and therefore, the results of all six studies are compiled together in Fig. 7.

Cultures of microglia that received no in vitro stimulation with P2C produced very low levels of cytokines. Microglial cultures stimulated with P2C only at 24 h (“stimulated protocol”) produced significant amounts of TNFα and IL-6. In contrast, microglia cultures stimulated with P2C at both 0 and 24 h (“tolerance protocol”) produced significantly less TNFα and IL-6 (Fig. 7a, b; Additional file 1: Fig. S1). These results indicate that microglia, as with BMDM, can be induced to become TLR2 tolerant. While TLR2 tolerance induction resulted in decreased production of the pro-inflammatory cytokines TNFα and IL-6, it had the opposite effect on the production of the regulatory cytokine IL-10. Although not quite reaching statistical significance, microglia stimulated with P2C at both 0 and 24 h (“tolerance protocol”) produced nearly twofold more IL-10 when compared with microglia stimulated only at 24 h (“stimulated protocol”) (Fig. 7c). Enhanced expression of IL-10 as a result of TLR tolerance induction has previously been documented in dendritic cells [29]. Overall, these in vitro results are consistent with our systemic in vivo TLR2 tolerance results described above in terms of TNFα production, confirming that isolated microglia manifest a less pro-inflammatory and more regulatory function when TLR2 tolerance is induced.

## Discussion

A growing body of evidence implicates TLR2 signaling as a key mechanistic factor in the pathogenesis of MS and EAE [10–12], and the potential role of the microbiome in the pathogenesis of MS further complicates the issue. We have previously reported that a microbiome-derived bacterial lipopeptide, Lipid 654, can be detected in the serum of all healthy individuals but is found at significantly lower levels in the serum of MS patients [16]. Based on this finding, we postulated that a normal function of the microbiome is to chronically expose the systemic innate immune system to low levels of microbial products which then serve to tolerize (i.e., regulate) innate responses. The homeostatic level of microbiome-mediated tolerance normally achieved is appropriate for responding to infectious agents without over-responding to endogenous TLR-signaling agonists (e.g., DAMPs), the latter potentially resulting in autoimmunity.

The concept of TLR tolerance induction is well established both in vitro and in vivo [20]. This phenomenon involves initial ligation and signaling through a TLR followed by significantly diminished responses upon subsequent stimulation through the same TLR or other related TLRs. This mechanism of tolerance has been shown to be mediated by the upregulation of miRNAs that mediate cytokine production [30], increased expression of the NFκB regulator A20 [31], and activation of intracellular regulatory molecules including IRAK-M that effectively diminish subsequent ligand-induced responses [20]. We have postulated that when microbiome-derived products (e.g., Lipid 654) do not access the systemic circulation in concentrations that are sufficient to induce an appropriate level of tolerance, the normal regulatory function of the microbiome is lost and innate immune responses are over-responsive and potentially self-reactive. The significantly lower level of serum Lipid 654 that we reported in MS patients suggested that the “normal” level of microbiome-induced tolerance and regulation of innate immune responsiveness is deficient in MS.

In proof of concept studies, we reported that repeated daily administration of low dose, non-inflammatory levels of a TLR2 ligand induced a systemic reduction in TLR2 signaling (“TLR2 tolerance”) and attenuated disease in a T cell transfer model of EAE [10]. In this study, TLR2 tolerance induction was associated with deficient activation of CNS antigen-presenting cells and significantly diminished CNS inflammation [10]. In a subsequent proof of concept study, we found that 50% of MS patients exhibited enhanced peripheral blood monocyte TLR2-stimulated responses, further suggesting deficient regulation of TLR2 responses in these patients [17]. These studies add confirmation to the underlying postulate that lack of sufficient circulating levels of microbiome-derived microbial products in MS results in defective systemic innate immune regulation and TLR2 over-responsiveness.

MS not only involves an autoimmune CNS inflammatory process, but also involves a defect in the ability to remyelinate the neurons demyelinated in this inflammatory process [3, 8, 9]. In addition to the controversial role of TLR2 in the inflammatory pathogenesis of MS and EAE, a small number of murine studies have also suggested that TLR2 signaling can inhibit remyelination. Prior studies reported that the in vitro maturation of OPC to OL is inhibited by TLR2 ligation and that hyaluronate, an endogenous TLR2 ligand (i.e., a DAMP), may be a relevant mediator of this inhibition of remyelination [15]. Furthermore, mice with a global deletion in TLR2 demonstrated enhanced remyelination in vivo after direct CNS administration of lysolecithin to induce demyelination [15], although these studies did not examine cuprizone-induced demyelination. Additional evidence suggesting an inhibitory role for TLR2 signaling in CNS remyelination include a study demonstrating that the TLR2 agonist, P3C, inhibited the pro-myelinating effects of erythropoietin [14], and a study reporting that cuprizone feeding resulted in increased expression of TLR2 in the CC and hippocampus [13]. Based on these prior studies of the potential involvement of TLR2 in the remyelination process, the lack of feasibility of a practical therapeutic approach for deleting TLR2 in vivo, and our EAE study suggesting the potential for a therapeutic reduction in TLR2 signaling using TLR2 tolerance induction [10], the goal in the present study was to test the role of TLR2 tolerance induction in the process of remyelination.

In the present study, mice were fed with cuprizone for 5 weeks to induce a non-inflammatory demyelination, followed by a 2-week feeding with normal chow to allow for remyelination. We asked whether diminishing TLR2 signaling by inducing systemic TLR2 tolerance during the 2-week recovery period would enhance remyelination. Our results showed that inducing TLR2 tolerance during the recovery period resulted in a significant enhancement in remyelination as documented by EM evaluation of both percentages of unmyelinated axons and myelin thickness (g-ratios).

We chose to focus on the 2-week post-cuprizone feeding time point to evaluate the effects of TLR2 tolerance induction on remyelination based on the well-documented time course of cuprizone-induced demyelination and recovery. We determined that 2 weeks post-cuprizone-cessation would provide the optimal window for assessing potential TLR2 tolerance-induced effects on remyelination. Numerous published studies have investigated the kinetics of remyelination after the cessation of cuprizone [32–34]. Importantly, these studies documented that complete remyelination, i.e., remyelination approaching control levels, is normally not seen for at least 4–5 weeks after cuprizone is stopped. In contrast, our results demonstrate that at the 2-week post-cuprizone time point, TLR2-tolerant mice demonstrate myelin metrics that are already statistically identical to control cohorts, i.e., mice that have never been exposed to cuprizone.

Using immunohistofluorescent approaches, we found that the TLR2 tolerance-induced enhancement in remyelination was not associated with changes in the number of OPCs or mature OLs, but rather was associated with a change in the balance of microglial phenotypes from pro-inflammatory iNOS^+^ to a non-inflammatory/pro-repair Arg1^+^ phenotype. Functionally, microglia (like macrophages) can be divided into different states of activation. M1 microglia are described as “pro-inflammatory” with regards to their cytokine secretion while M2 microglia (“alternatively activated” or “anti-inflammatory” microglia) are associated with extracellular matrix remodeling, progenitor cell differentiation, and tissue regeneration [24, 25, 35]. It has become clear that the M1/M2 paradigm is too simplified to describe the complexity of microglia in vivo. The terms pro-inflammatory and anti-inflammatory more accurately describe the significant in vivo functional differences associated with iNOS^+^ Arg1^−^ versus iNOS^−^ Arg1^+^ microglia.

One limitation of our finding is that we have based our in vivo microglial phenotypic characterization on iNOS versus Arg1 expression rather than a more broad characterization at the transcriptome level. However, it should be noted that important aspects of macrophage and microglial biology are fundamentally driven by the phenotype of arginine metabolism utilized and, consistent with the functional in vivo differences in these microglia, pro-inflammatory and anti-inflammatory/pro-repair cells metabolize arginine differently [36]. Pro-inflammatory cells utilize the enzyme nitric oxide synthase to metabolize arginine to nitric oxide and citrulline while anti-inflammatory/pro-repair cells utilize arginase to hydrolyze arginine to ornithine and urea. Importantly, both of these arginine metabolic pathways cross-inhibit each other [36].

Our results suggest that the enhanced remyelination seen with TLR2 tolerance is mediated, at least in part, by effects on microglial phenotype, and specifically on an increase in the percentage of iNOS^−^ Arg1^+^ microglia. “M2” polarization of microglia has been documented to result in enhanced remyelination, and while there is not yet a clear understanding of how M2 microglia mediate enhanced remyelination, proposed mechanisms include increasing OPC to OL differentiation [26, 37] and increasing phagocytic clearance of myelin debris [38, 39]. In addition to our in vivo analysis, we also used an in vitro approach, confirming that microglia are easily induced to become TLR2 tolerant following a single stimulation with a TLR2 agonist. Moreover, in vitro TLR2-tolerized microglia demonstrated reduced secretion of pro-inflammatory cytokines and increased the production of the immune-regulatory cytokine IL-10. These results are in agreement with our previous in vitro studies of murine BMDMs [10] and in agreement with studies using primary rat microglial cultures induced to TLR4 tolerance using LPS [40].

Finally, with the goal of confirming that the enhanced remyelination documented in P2C-treated mice was a result of TLR2 tolerance induction and not related to repeated low dose TLR2 stimulation, we performed remyelination studies utilizing TLR2^−/−^ mice. In confirmation of our TLR2 tolerance results, we documented that TLR2^−/−^ mice show a significant enhancement in remyelination during recovery from cuprizone exposure. Studying mice with a global deletion of TLR2 precludes our commenting on the mechanistic role of microglia in the enhanced remyelination observed in these mice. To this end, future studies will utilize mice with microglial-specific deletion of TLR2.

In addition to OPC, OL, and microglia, astrocytes have been described as having both an enhancing and inhibitory effect on remyelination. As reviewed by Alizadeh et al. [41], astrocytes have been documented to play a role in clearance of myelin debris, modulating OL activity through intercellular connections, production of OL growth factors, and secreting chemokines that recruit cells, including microglia, into sites of demyelination. However, astrocytes also form glial scars which play an inhibitory role in CNS remyelination in part through the secretion of chondroitin sulfate proteoglycans (CSPGs) and other moieties which limit the ability of OPCS to migrate and differentiate into mature myelinating OLS [41]. In the present study, we have not specifically examined the effects of TLR2 tolerance induction on astrocytes but future studies will not only focus on mice with microglial-specific TLR2 deletion, but also on mice with astrocytic-specific deletion of TLR2.

The present study represents the next phase of proof-of-concept studies in which we investigate the use of TLR2 tolerance induction as a therapeutic approach in MS and potentially in other neuroinflammatory diseases as well. Unlike total genetic deletion of TLR2 (which would not be feasible in humans), the induction of TLR2 tolerance allows for regulation of the level of TLR2 responsiveness. Thus, our approach does not completely eliminate TLR2 signaling (e.g., see Fig. 1c), but rather “rheostats” it down to a level that we believe is significantly less pathogenic when receiving the relatively weak signals from endogenous ligands such as DAMPs. Importantly, in regard to the feasibility of applying this approach in humans, using careful titration, an appropriately low dose of TLR2 ligand(s) could be chosen that would induce tolerance without inducing pro-inflammatory signs or symptoms. This is the approach we have used to determine the TLR2 tolerizing dose of P2C administered in our studies. In mice, after the administration of the first low dose of P2C, which results most often in either no clinical effect or, at most, in a very mild hunching posture, all subsequent doses of P2C (with tolerance now induced) elicit no clinical signs. A similar approach is theoretically feasible in humans as well. Thus, we believe that “TLR2 tolerant” individuals will manifest no signs of pro-inflammatory effects from the low dose administration of ligands, but importantly will demonstrate a level of systemic TLR responsiveness that is diminished from pre-tolerance levels, while still allowing for appropriate responsiveness to the strong ligand signals presented by infectious organisms.

## Conclusion

The present study presents evidence that the innate immune system, and TLR2 specifically, has a role in the process of remyelination. Moreover, together with our previous findings of lower serum Lipid 654 levels in MS patients [16], TLR2 tolerance-induced attenuation of EAE [10], and enhanced TLR2 responsiveness among MS patients [17], the present results represent further proof-of-concept evidence that inducing TLR2 tolerance in vivo mediates functional changes in TLR2 signaling that has physiologic and disease-relevant effects. By demonstrating the relevance of TLR2 signaling in remyelination, the present study also confirms earlier studies suggesting that TLR2 signaling plays a critical role in the defect in remyelination noted in patients with MS. Collectively, the present results and our previous EAE study suggest that induction of TLR2 tolerance may represent a novel two-pronged approach to MS treatment, inhibiting autoimmune inflammation while simultaneously facilitating myelin repair.

## Additional files

Additional file 1: **Figure S1.** Microglia FACS-sorting strategy. Non-adherent cells (microglia) from 12 to 18 day mixed glial cultures were harvested, antibody stained, and purified by FACS-sorting for CD45.2^+^ CD11b^+^ microglia. The gating strategy used and the pre- and post- sort outcomes are depicted. (JPG 205 kb)

Additional file 2: **Table S1.** Cytokine levels in microglial culture supernatants. 96-well microglial cultures were designated as either “non-stimulated”, “stimulated” or “tolerized”. “Non-stimulated” wells received no stimulation over the entire 2 days of culture. “Stimulated” wells received 1 μg/mL P2C at the 24 hour time point. “Tolerized” wells received 1 μg/mL P2C both at the initiation of culture and at the 24 hour time point. After 48 hours, supernatants were harvested and assayed for cytokines via multiplex enzyme-linked immunosorbent assay (ELISA). Mean values are calculated from 2 replicate ELISA determinations and represent pg/ml. (JPG 463 kb)

## Data Availability

The datasets used and/or analyzed during the current study are available from the corresponding author on reasonable request. The g-ratio analysis plug-in and source code for the ImageJ software (http://rsbweb.nih.gov/ij), which allowed for semi-automated analysis of randomly selected sets of fibers, are available online (http://gratio.efil.de).

## Abbreviations

ANOVA: Analysis of variance
Arg1: Arginase 1
CC: Corpus callosum
CC1: Mouse monoclonal antibody marketed as anti-adenomatous polyposis coli clone CC1
CD: Cluster of differentiation
CNS: Central nervous system
DAPI: 4′,6-diamidino-2-phenylindole
EAE: Experimental autoimmune encephalomyelitis
ELISA: Enzyme-linked immunosorbent assay
EM: Electron microscopy
i.v.: Intravenous
IBA1: Ionized calcium binding adaptor molecule 1
IL-10: Interleukin 10
IL-6: Interleukin 6
iNOS: Inducible nitric oxide synthase
MS: Multiple sclerosis
ns: Not significant
OL: Oligodendrocyte
OPC: Oligodendrocyte precursor cell
P2C: Pam2CSK_4_, a synthetic diacylated lipopeptide
P3C: Pam3CSK_4_, a synthetic triacylated lipopeptide
PBS: Phosphate-buffered saline
PDGFRα: Platelet-derived growth factor receptor α
PFA: Paraformaldehyde
RRMS: Relapsing-remitting multiple sclerosis
SPMS: Secondary progressive multiple sclerosis
TEM: Transition electron microscope
TLR2: Toll-like receptor 2
TLR2^−/−^: Toll-like receptor 2-deficient
TNFα: Tumor necrosis factor α
VC: Vehicle control
WT: Wild-type
