# Supplementary figures and images for: Systemic TLR2 tolerance enhances central nervous system remyelination
Source: J Neuroinflammation. 2019 Jul 27;16:158. doi: 10.1186/s12974-019-1540-2 (PMC6660683; doi:10.1186/s12974-019-1540-2)

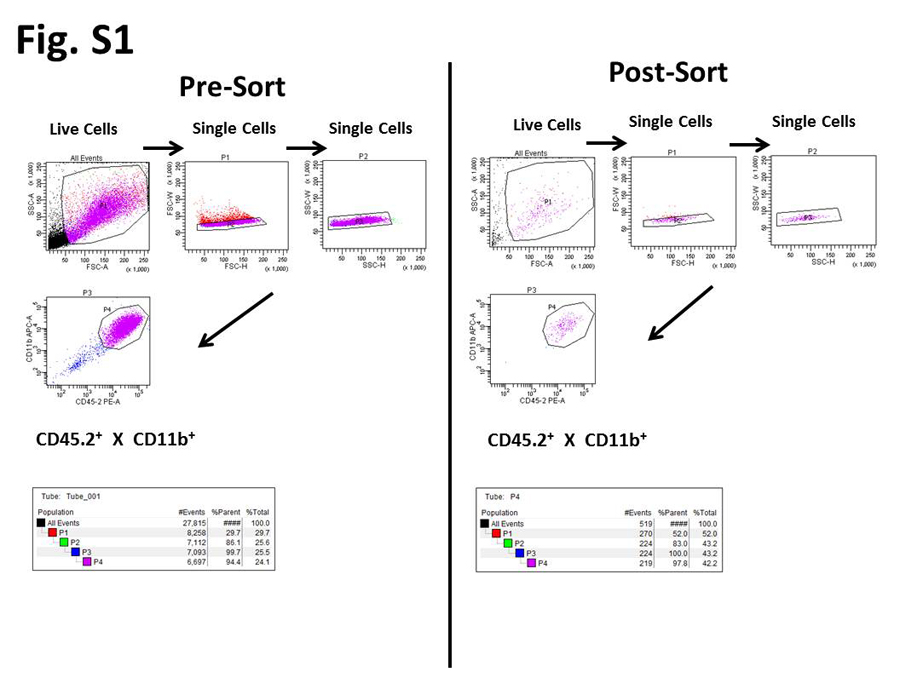

Supplement: Supplementary file 1 — Figure S1. Microglia FACS-sorting strategy. Non-adherent cells (microglia) from 12 to 18 day mixed glial cultures were harvested, antibody stained, and purified by FACS-sorting for CD45.2+ CD11b+ microglia. The gating strategy used and the pre- and post- sort outcomes are depicted. (JPG 205 kb) [file 12974_2019_1540_MOESM1_ESM.jpg]

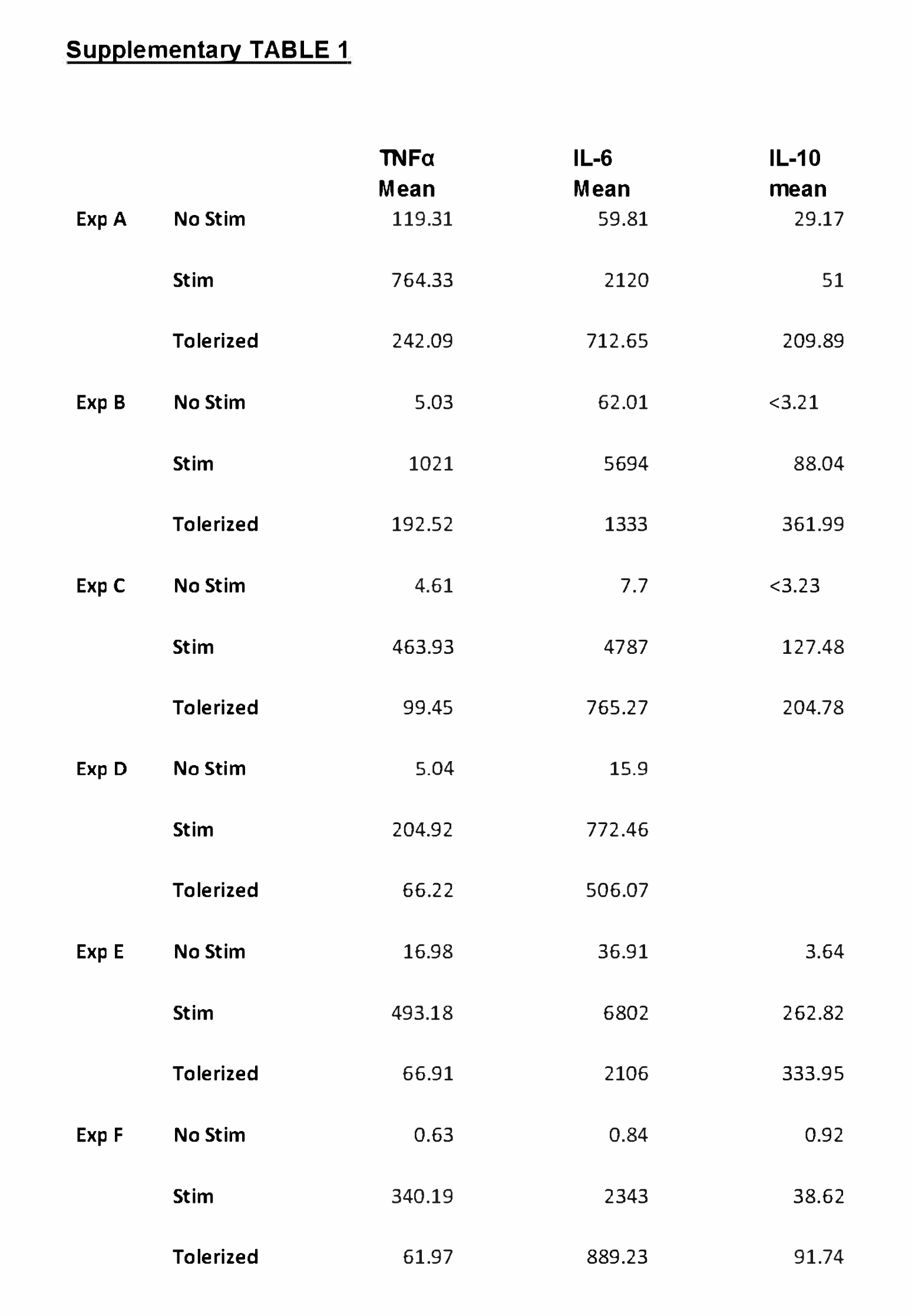

Supplement: Supplementary file 2 — Table S1. Cytokine levels in microglial culture supernatants. 96-well microglial cultures were designated as either “non-stimulated”, “stimulated” or “tolerized”. “Non-stimulated” wells received no stimulation over the entire 2 days of culture. “Stimulated” wells received 1 μg/mL P2C at the 24 hour time point. “Tolerized” wells received 1 μg/mL P2C both at the initiation of culture and at the 24 hour time point. After 48 hours, supernatants were harvested and assayed for cytokines via multiplex enzyme-linked immunosorbent assay (ELISA). Mean values are calculated from 2 replicate ELISA determinations and represent pg/ml. (JPG 463 kb) [file 12974_2019_1540_MOESM2_ESM.jpg]
